# Gender Differences in Behçet's Disease Associated Uveitis

**DOI:** 10.1155/2014/820710

**Published:** 2014-04-23

**Authors:** Didar Ucar-Comlekoglu, Austin Fox, H. Nida Sen

**Affiliations:** ^1^National Eye Institute, National Institutes of Health, Bethesda, MD 20892, USA; ^2^Ophthalmology Department, Cerrahpasa Medical Faculty, Istanbul University, 34080 Istanbul, Turkey; ^3^College of Medicine, University of South Alabama, Mobile, AL 36688, USA

## Abstract

Behçet's disease is a systemic vasculitis of unknown etiology, characterized by oral and genital ulceration, skin lesions, and uveitis as well as vascular, central nervous system, and gastrointestinal system involvement. It is prevalent in the Middle East, Mediterranean, and Eastern Asia. The aim of this review is to evaluate the gender differences in clinical manifestations of Behçet's disease, treatment responses, mortality, and morbidity. Behçet's disease has been reported to be more prevalent in males from certain geographic regions and particular ethnic groups; however, recent reports indicate more even gender distribution across the world. There are gender differences in clinical manifestations and severity of the disease. Ocular manifestations, vascular involvement, and neurologic symptoms are more frequently reported in male patients whereas oral and genital ulcers, skin lesions, and arthritis occur more frequently in female patients. The disease can have a more severe course in males, and overall mortality rate is significantly higher among young male patients.

## 1. Introduction

Behçet's disease (BD) is a rare immune-mediated small vessel systemic vasculitis of unknown etiology. It is a multisystem disorder that presents with episodes of mucocutaneous lesions, uveitis, arthritis, venous thrombosis, arterial aneurysms, intestinal ulcers, pulmonary lesions, and central nervous system lesions. Between episodes, clinical findings may be completely normal [1]. BD predominantly affects people with lineages from the Silk Road, particularly Turkey and Japan. BD is more prevalent in certain geographic regions and among particular ethnic groups. There is a strong association with HLA-B51 as has been confirmed by recent genome-wide association studies [2]. Studies have also indicated that shared risk loci with other autoimmune and autoinflammatory diseases, such as ankylosing spondylitis, inflammatory bowel disease, and familial Mediterranean fever, implicate shared and complicated pathogenic pathways involving both the innate and adaptive immune system in Behçet's disease [3–5]. HLA-B51 has been shown to be present in 40–70% of patients from the Middle East and Asia; however, it is found in only 13% of patients in Europe and North America [6]. Patients with HLA-B51 have a sixfold increased risk of BD, and the disease is usually more severe in HLA-B51 positive patients [4]. Familial BD has been reported in 1–18% of patients, mostly in Turkish, Israeli, and Korean populations [6].

Patients often present in their 30 s–40 s with recurrent oral aphthous ulcers, genital ulcers, and uveitis [7]. Children are rarely affected [5]. In contrast to early reports of higher male to female ratios from Turkey and Japan [8–10], this ratio is now nearly equal with the only exception being Arab countries where higher male prevalence persists. A recent large Chinese population-based study showed no significant gender difference in the incidence or prevalence of Behçet's disease [11, 12].

BD exhibits a more severe course in males as well as in patients with younger age of onset and HLA-B51 positivity [13]. According to the International Study Group (ISG) for Behçet's disease diagnostic guidelines, the patient must have recurrent oral (aphthous) ulceration (at least three times within a 12-month period) along with 2 out of the following 4 symptoms: recurrent genital ulcers, ocular inflammation (anterior and/or posterior uveitis, cells in the vitreous, and retinal vasculitis), skin lesions (including erythema nodosum, pseudofolliculitis, papulopustular lesions, and acne in postadolescents not on corticosteroids), and positive pathergy test [14]. Each of these clinical manifestations may affect men and women differently (Table 1).

## 2. Gender Differences in Extraocular Manifestations of Behçet's Disease

Mucocutaneous lesions are the most frequently observed findings of BD and include oral and genital ulcers, acneiform lesions, papulopustular lesions, erythema nodosum-like lesions, and superficial thrombophlebitis. Cutaneous lesions constitute part of the major criteria for the diagnosis. The most frequent cutaneous manifestations are erythema nodosum-like lesions, papulopustular lesions, erythema multiforme, and extragenital ulcerations. Erythema nodosum-like lesions are more frequently seen in females and typically affect the lower limbs. These lesions usually resolve within 2-3 weeks with residual pigmentation [15, 16]. Papulopustular lesions are more frequently seen in males [15–17].

**Figure 1:**
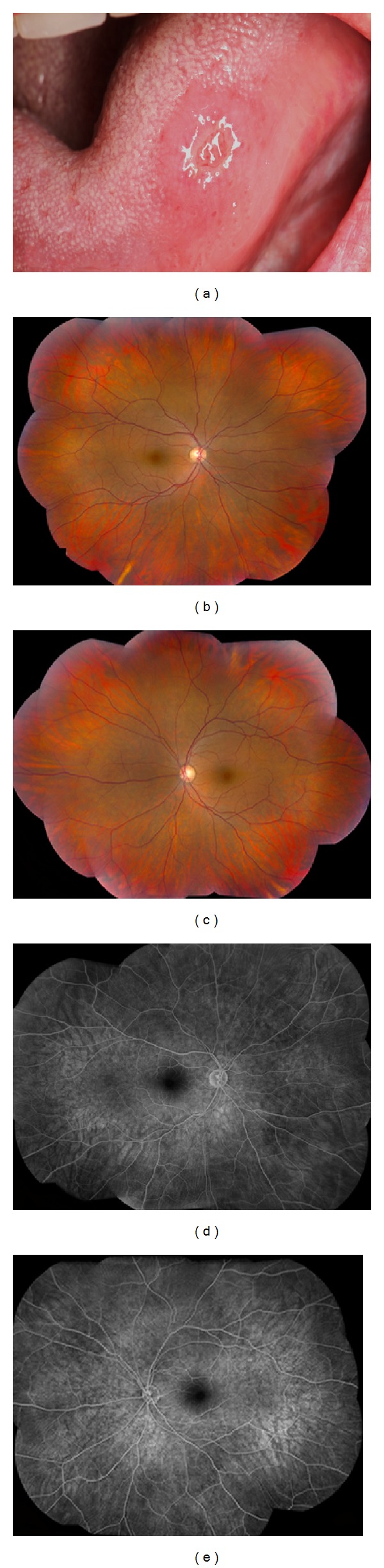
A 36-year-old Iranian female with incomplete Behçet's disease with history of oral ulcers (a), genital ulcers, and nongranulomatous anterior uveitis. Retinal exam was completely normal with a visual acuity of 20/16 in each eye. Fundus photos ((b) and (c)) and fluorescein angiogram ((d) and (e)) confirm the absence of retinal vasculitis and retinitis ((b)–(e)).

Oral aphthous ulcers are typically recurrent painful ulcerations of the oral mucosa that last up to 14 days. Oral ulcer is the most common manifestation of BD (found in 95–100% of patients) and can be the presenting manifestation in about 70% of patients [12, 18–20]. According to ISG for BD criteria, oral ulcers recurring at least three times over a 12 month period are crucial to the diagnosis [14]. In a retrospective review of 3527 BD patients, Oh et al. found that oral ulcers were more common in females and exacerbations correlated with menstrual cycles [21] (Figure 1(a)).

Genital ulcers are less likely than oral ulcers to recur, often heal with scarring, and can be found in 62% to almost 100% of BD patients [12, 22, 23]. Similar to oral ulcers, genital ulcers are also more frequent in females [11, 21, 24–28]. In males, scrotum is more likely to be involved whereas in females, genital ulcers are frequently seen on labia majora and minora [29]. Genital ulcers are especially common and larger in females with BD and resemble recurrent aphthous stomatitis [15].

The skin pathergy test is a skin hyperreactivity test induced by a needle prick. Typically, papule formation (>2 mm diameter), ≥24–48 hours following a sterile needle prick to the forearm, is considered a positive response [14]. According to the ISG for BD, pathergy positivity is among the major criteria for the diagnosis. Different pathergy reaction rates have been reported worldwide (6–71%) [30], but it is especially high in Japan (44%) [31] and the Middle East (60–70%) [32]. Pathergy positivity is more common in males [15, 16] but is not associated with an increased risk for specific mucocutaneous or systemic involvement or a more severe disease course. An epidemiologic study from Korea reported an overall female predominance among BD patients and higher positivity of pathergy test in male patients [33].

Arthritis and arthralgia have been reported in approximately 35–50% of BD patients [34]. Some reports revealed high frequencies in females (56%) [24, 35] whereas some reports indicated similar incidence in both sexes [26]. It is usually mono- or oligoarticular arthritis and typically resolves in a few weeks without deformity or radiological erosions. The knee joint is the most commonly affected followed by ankle, wrist, and elbow joints. Joint manifestations are frequently seen with erythema nodosum and thrombophlebitis and seem to be more common in patients with papulopustular lesions [22, 32].

Vascular involvement can occur in 7.7% to 43% of patients. Even though vasculitis is a significant feature of Behçet's disease, it is not one of the ISG diagnostic criteria. Both veins and arteries of all sizes can be affected with an associated thrombotic tendency [1, 10, 36–41]. Venous involvement is more common than arterial (88% versus 12%) [39]. Venous thrombosis is the most common vascular manifestation occurring in 6.2% to 33% [42, 43]. Vascular involvement in BD is more common in males and has a more severe course [37]. A review of 137 Turkish BD patients showed vascular involvement in 27.7% with venous involvement in 24% and arterial involvement in 3%. Vascular involvement was more common in males with a male to female ratio of 4.4 : 1. Additionally, ocular involvement and pathergy positivity were significantly more common among patients with vascular disease [39]. Similarly, a subsequent study of 2,147 Turkish patients with BD also showed that male patients were five times more likely to have vascular complications [10].

Central nervous system (CNS) involvement (*neuro-Behçet's*) occurs in approximately 5% of patients and is one of the most serious causes of long-term morbidity and mortality [44–47]. Neuro-Behçet's is more prevalent in males with a male to female ratio of 2 : 1 to 3 : 1. In a large retrospective study from Turkey, the frequency of CNS involvement was 13% in men and 5.6% in women. Among 200 Turkish neuro-Behçet's patients, the male to female ratio was 3.4 : 1. Other groups from Iraq, Tunis, and Italy have also reported higher rates of neuro-Behçet's in males with a male to female ratio ranging from 1.6 : 1 to 2.8 : 1. In these studies male gender and CNS involvement were also found to be associated with a poor prognosis [46–51]. The age of onset of neuro-Behçet's is generally 20–40 years, though it has been reported in children [52]. Neurological signs commonly develop a few years after the onset of the other systemic manifestations of BD [46, 50].

Intestinal involvement is rare but can be a common cause of mortality and severe morbidity in BD [53]. Gastrointestinal (GI) manifestations of BD usually occur 4.5–6 years after the onset of oral ulcers. The prevalence of GI involvement is higher (50–60%) in Japan and Korea, while it is much lower in Turkey and Israel (0–5%). The frequency of extraoral GI involvement varies widely among different ethnic groups [25, 53, 54]. Although several studies reported no gender difference in the incidence of GI involvement, male predominance has been reported by some [11, 25, 55]. Ulcerations may occur anywhere from the mouth to the anus in the GI tract; however, the ileocecal region with extension into the ascending colon is the most frequent site of extraoral involvement [56].

## 3. Gender Differences in Prevalence, Incidence, and Severity of Behçet's Disease Associated Uveitis

**Figure 2:**
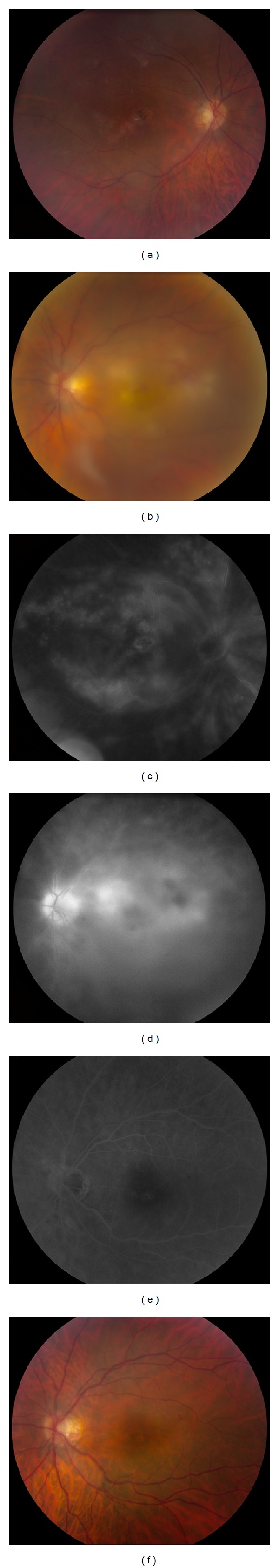
A 30-year-old Jewish male presented with panuveitis, retinitis, and retinal vasculitis and was later diagnosed with Behçet's disease. Right eye was legally blind with a visual acuity of 20/200 due to a macular retinitis in the past. Left eye visual acuity was 20/640 due to active macular retinitis. Fundus photos and fluorescein angiogram show macular scar (a) in the right eye and active macular retinitis in the left eye (b) with diffuse retinal vascular leakage in both eyes (involving both veins and arteries) and late staining of the retinitis in the left eye ((c) and (d)). Left eye visual acuity improved to 20/50 after treatment with infliximab with resolution of retinitis and retinal vasculitis ((e) and (f)).

Uveitis in BD (BDU) has been reported in approximately 50% of the patients in multidisciplinary centers and more than 90% in ophthalmology reports [4, 57]. Patients usually present with bilateral nongranulomatous panuveitis and retinal vasculitis. However, it may rarely present as isolated anterior uveitis, particularly in female patients [58] (Figure 1). Episcleritis, scleritis, conjunctival ulcers, keratitis, orbital inflammation, isolated optic neuritis, and extraocular muscle palsies are rare forms of ocular involvement [59]. Uveitis occurs within 3–5 years after the onset of BD; however, ocular manifestations may be the initial manifestation in approximately 10–20% of cases [58, 59]. Similar to vascular and neurologic involvement, BDU is also more common in males [10]. Typically, BDU has a relapsing and remitting course with explosive episodes and quiet periods in between. Sudden onset of uveitis flare-ups and spontaneous resolution are important features of the disease [58]. Anterior uveitis (iridocyclitis) with hypopyon is very characteristic but occurs in only 10–30% of patients. Hypopyon in BD forms a smooth layer and shifts with head positioning. Posterior synechiae, peripheral anterior synechiae, iris atrophy, cataract, and secondary glaucoma can be seen as complications of recurrent anterior uveitis attacks [58]. Anterior uveitis and hypopyon occur more commonly in women whereas panuveitis and severe ocular BD are more common in men [59, 60] (Figure 2). Childhood onset BDU is also more common in males [61].

Posterior uveitis patients present with decreased vision with floaters and/or visual field defects. Diffuse vitritis, retinal infiltrates, sheathing of retinal veins, occlusive vasculitis, swelling of the optic disc, branch retinal vein occlusions, and exudative retinal detachment are common posterior segment findings. The classic posterior uveitis finding is retinal vasculitis, which can affect both arteries and veins. Retinal disease is the most serious form of ocular involvement in BD [58]. Maculopathy and optic atrophy are the most common causes of permanent visual loss [17]. Optic disc involvement can occur in the form of acute anterior neuropathy, papilledema as a result of dural sinus thrombosis or benign intracranial hypertension, neuroretinitis, or retrobulbar optic neuropathy [46, 62].

In a large retrospective study from Turkey, the mean age at onset of uveitis was 28.5 years in males and 30 years in females. Bilateral ocular involvement was seen in 78.1%. There was no gender difference in terms of bilaterality and recurrence of uveitis [61]. However, panuveitis was more common in male patients [63]. Sight-threatening fundus lesions and complications were also more common in male BD patients [61]. In a study by Tugal-Tutkun et al., hypopyon, vitritis, retinal vasculitis, retinitis, and retinal hemorrhages were also seen more frequently in male patients while papillitis was more common in females [61]. According to some reports, male patients with BDU have worse visual prognosis likely due to the fact that men with BD are more likely to have panuveitis [17, 61].

## 4. Gender Differences in Treatment and Prognosis of Behçet's Disease

There may be gender differences in response to treatment as well. Mat et al. reported that methylprednisolone acetate was effective for erythema nodosum in females but not in males [64]. Similarly, Yurdakul et al. reported in 116 BD patients from Turkey that colchicine had favorable effects on genital ulcers, erythema nodosum, and arthritis in females but only for arthritis in males [65]. Hamuryudan et al. showed that different dosages of thalidomide were effective for oral and genital ulcers and follicular lesions in male patients; however, the study did not assess thalidomide's effect in females because of its teratogenic effects [66]. Another male-only study reported that azathioprine 2.5 mg/kg daily was effective for the preservation of visual acuity and the prevention of incident ocular BD as well as mucocutaneous lesions and arthritis [67]. Masuda et al. reported cyclosporine 10 mg/kg daily to be more effective than colchicine 1 mg daily for the treatment of ocular disease and oral and genital ulcers in a double-masked randomized trial [68]. Interestingly, treatment side effects also differed between males and females; hirsutism with cyclosporine occurred more commonly in females while neurotoxicity was significantly more common among males [68, 69].

Several reports showed that the overall mortality rate in BD is significantly higher among male patients. The rate is especially high for young males in their 20 s–40 s [47, 70]. Common causes of mortality are pulmonary arterial aneurysms and neurological involvement, which are significantly more common among young male patients [4].

## 5. Possible Explanations for Gender Differences in Behçet's Disease

Autoimmune diseases tend to be more common in women of childbearing age. However, Behçet's disease is equally prevalent among males and females in some geographic regions and more prevalent in males in others [4, 34]. Overall, the disease has a more severe course and higher mortality among male patients [47]. Despite numerous studies indicating notable gender differences in ocular and extraocular manifestations as well as severity and mortality of the disease, there is no clear evidence as to what this difference stems from. Although its etiology is unknown, both genetic and environmental factors (smoking, infection, vitamin D, and immune dysregulation) have been blamed [1, 13]. Whether males are more prone to such environmental risk factors has yet to be determined. Both smoking and cessation of smoking have been implicated in severity of clinical manifestations of BD including vasculitis and mucocutaneous lesions [71–73]. Smoking was more common among male patients with BD in some studies raising the question of possible association [71–73]. Similarly, low vitamin D3 levels have been associated with BD or its severity; however, these studies failed to show significant differences in vitamin D3 levels between male and female BD patients [74, 75]. Both male gender and HLA-B51 have been consistently associated with a severe disease course and poor prognosis in BD. In fact, a recent meta-analysis study indicated that HLA-B51 was more common among male BD patients [76], suggesting there could be a genetic basis for poor prognosis among men with BD. While most other autoimmune diseases are more common among women of childbearing age, BD seems to differ with either equal gender distribution or male predominance. The relationship between BD and pregnancy is also poorly studied. Effects of pregnancy on Behçet's disease in 27 patients showed worsening of disease in 2/3 of patients during pregnancy, particularly in the 1st trimester. This same group also noted exacerbations in oral and genital ulcers during premenstrual periods. These findings suggest that progesterone may play a role in the disease course among women in a complex manner [77]. Whether other reproductive or sex hormones play any role or to what extent has yet to be determined.

## 6. Conclusion

In summary, mucocutaneous involvement is the hallmark of BD and is more common in females. Neurologic involvement and major vessel disease are uncommon, but such involvements are life-threatening and more common in males. Ocular BD is more common in males whereas arthritis is more frequently reported in female patients. Male patients are more likely to be affected at a younger age, have a more severe uveitis, present with worse visual acuity, and suffer vision loss over time [4, 58]. Though bilaterality and recurrence of uveitis are similar between sexes, incidence of panuveitis and vision loss are higher in men [29]. Overall mortality rates are also higher in young male patients [47]. Despite gender differences in severity of ocular and extraocular findings, visual prognosis in BDU has improved over the past decade due to more aggressive use of immunomodulatory therapy [34].

It is intriguing as to why BD is more prevalent, at least in some reports, and more severe among men when most autoimmune diseases are more prevalent or more severe among women [11]. Although some of the aforementioned risk factors may indeed be responsible for more severe disease in men, there are, unfortunately, no studies directly evaluating possible reasons for gender differences in BD. Translational and epidemiologic studies are needed to further address the question of gender in Behçet's disease.

## Figures and Tables

**Table 1:** Behçet's disease and gender differences in clinical manifestations.

Clinical findings	Incidence/prevalence	Severity*
Mucocutaneous lesions	Erythema nodosum more common in females Papulopustular lesions more common in males	Comparable
Oral ulcers	More in females	Comparable
Genital ulcers	More in females	More severe in females
Skin pathergy test	More in males	Comparable
Arthritis and arthralgia	More in females**	Comparable
Vascular involvement	More in males	More severe in males
Central nervous system involvement	More in males	More severe in males
Gastrointestinal manifestation	Comparable	Comparable
Uveitis	More in males (*anterior uveitis is more common in women*;* panuveitis is * *more common in men*)	More severe in males

*Comparable indicates that the severity of these clinical manifestations were not significantly different in most studies.

**Some studies indicated arthritis to be more common in females while others showed comparable incidence.
